# Differential coexpression network modules observed in human hepatocellular carcinoma progression

**DOI:** 10.1186/1471-2105-14-S17-A11

**Published:** 2013-10-22

**Authors:** Hui Yu, Zhongming Zhao

**Affiliations:** 1 Department of Biomedical Informatics, Vanderbilt University School of Medicine, Nashville, TN 37203, USA; 2 Department of Psychiatry, Vanderbilt University School of Medicine, Nashville, TN 37232, USA; 3 Department of Cancer Biology, Vanderbilt University Medical Center, Nashville, TN 37232, USA

## Background

While an understanding of the human interactome is within attainable reach [1], an impending challenge is to uncover the condition-specific dynamics of the protein-protein interaction (PPI) network, especially those that coordinate with disease progression [2]. Differential coexpression analysis (DCA) [3,4] has recently emerged as an effective approach to address this issue, but such an effort has yet to be thoroughly tested.

## Materials and methods

In this work, we explored the validity of extracting context-specific PPI subnetworks by analyzing the differential coexpression of interacting protein pairs. We first compared highly differentially coexpressed genes (“high-DC genes”) with highly differentially expressed genes (“high-DE genes”) in terms of their fit within a PPI-network analysis. Then, in a human PPI network overlaid with gene-gene differential correlation (DC) values calculated from the microarray gene expression dataset GSE6764, we sought high-DC subnetworks for each disease stage transition of hepatocellular carcinoma (HCC).

## Results

The validity of integrating DCA with a PPI network was demonstrated in two lines of evidence. First, higher expression correlations were associated with PPI pairs than non-PPI pairs, and exceptionally high DC values were observed within part of the PPI pairs. Second, compared with the high-DE genes, high-DC genes were more enriched with HCC-related genes and were more condensed in the reference network. Then, we extracted DC-PPI subnetworks for the four transitions over five HCC stages. All subnetworks turned out to be significantly enriched with HCC-related genes, HCV-targeted genes, and cancer genes. A comparison of the multiple transition-wise subnetworks gene by gene enabled us to identify the recurrent hub proteins, while comparing them edge by edge allowed us to identify protein pairs with constantly-changing relationships.

## Conclusions

We demonstrated a differential coexpression workflow within the context of a human PPI reference network. As applied to a multi-stage HCC expression dataset, our approach has generated a set of differentially coexpressed genes and network modules with promising candidates for follow-up HCC investigation.
